# Editorial: Detection and characterization of gastrointestinal (early) cancer

**DOI:** 10.3389/fonc.2022.1089612

**Published:** 2022-11-21

**Authors:** Andrej Wagner, Simona Gurzu, Valeria Barresi, Yuming Jiang

**Affiliations:** ^1^ Department of Internal Medicine I, University Clinics Salzburg, Paracelsus Medical University, Salzburg, Austria; ^2^ George Emil Palade University of Medicine, Pharmacy, Sciences and Technology of Târgu Mureş, Târgu Mures, Romania; ^3^ Department of Diagnostics and Public Health, University of Verona, Verona, Italy; ^4^ Department of Radiation Oncology, Stanford University School of Medicine, Stanford, CA, United States

**Keywords:** neoplasia, endoscopy - methods, invasion depth diagnosis, biomarker, characterization

Gastrointestinal carcinomas have the highest incidence and mortality worldwide (1). In this collection consisting of 26 original research articles, two reviews and a clinical case report, we focus on endoscopic characterization of gastrointestinal neoplasias (GN) and the emerging role of circulating biomarkers, commonly known as liquid biopsies. In addition, we present articles on histopathological markers, which may be useful for decision making after endoscopic resection.

Chromoendoscopy with Lugol’s solution is an essential technique for detection and delineation of SCC. Unfortunately, the specificity of this method is low (2). Guo et al. present a clinical study on factors leading to misdiagnosis of SCC after iodine staining. After multivariate analysis, they identified five risk factors independently associated with endoscopic misdiagnosis; these may be used to predict the probability of false positive results and to enhance specificity. Chen et al. also focus on the issue of multiple unstained areas in Lugol chromoendoscopy. In their retrospective analysis of 329 patients, they demonstrated that the endoscopic resection of a primary early SCC lesion combined with radiofrequency ablation of multiple Lugol-voiding lesions significantly reduces metachronous SCC lesions and low-grade dysplasia in the treated areas.

Apart from invasive screening and characterization, the role of non-invasive markers for detection of SCC is emerging. Metabolomics is a promising technology to characterize pathological processes and to explore potential biomarkers for cancer development. Yu et al. explored the metabolic profile of SCC at different stages compared with healthy controls. They concluded that SCC development is accompanied by persistent abnormal changes in oxidative stress. However, they could not identify any specific marker able to differentiate between early and invasive stages of the disease.

Tumors can induce changes in the behavior of platelets (3, 4). In the context of SCC, Liu et al. performed RNA sequencing of tumor-educated platelets to derive an RNA signature to distinguish ESCC patient samples from controls. They constructed a 3-gene platelet RNA signature that could differentiate early SCC from healthy controls with a sensitivity of 87.5% and a specificity of 81.1% in the validation cohort. However, the authors could not exclude smoking, geographic and ethnical differences as confounding factors in this study.

Several articles focus on biomarkers in gastric carcinoma (GC) progression and therapy. Zhang et al. identified Glutathione peroxidase 8 immunohistochemical expression as a negative prognostic marker in 83 patients. Jin et al. report fascinating insights in the role of a cancer testis antigen, Melanoma-associated antigen-A3, which is overexpressed in GC and may be linked to poor response to immunotherapy and poor prognosis. Quiao and his study group from Beijing (Qiao et al.) present results from their study on the role of the *CDKN2A* in apoptosis, cell cycle arrest and senescence in GC, showing that the inactivation of this gene could be a frequent causal factor and useful predictor for hematogenous spread of GC.

The characterization and risk stratification of gastric lesions before endoscopic resection or surgery could support therapeutic decisions and therefore improve clinical outcomes. In a large retrospective analysis, He et al. compare the prognostic values of different indices and tumor markers in GC at stages I-II. They outline the prognostic value of the systemic immune-inflammation index on patient survival, and propose an interactive web dynamic nomogram to use in clinical practice.

Radiomics is an emerging method combining quantitative features of multimodal imaging techniques and machine learning. Yang et al. used a radiomics model on dual-energy CT to improve accuracy for predicting serosal invasion in GC. This approach led to a high accuracy compared with traditional CT imaging (AUC of the testing set: 0.93 vs. 55.9%–90.8%, respectively (5).

Meticulous magnifying endoscopic evaluation has a high accuracy in detection of early gastric cancer (EGC). However, the distinction between EGC after eradication of H. pylori and gastritis may be challenging even for expert endoscopists (6). Furthermore, synchronous (pre-)malignant lesions are observed in up to 20%. Kurumi et al. report on the diagnostic yield of 5-aminolevulinic acid (5-ALA)-mediated photodynamic diagnosis in a cohort of 43 patients. In comparison with white light magnifying endoscopy, this approach could provide additional diagnostic yields to detect multiple lesions simultaneously.

We conclude the section on gastric cancer with a review on detection and endoscopic characterization of early GC by Ferreira et al.

In the context of colorectal cancer (CRC) screening by multitarget stool DNA methylation tests (7), Ma et al. present a novel stool test for the non-invasive screening of gastric and colorectal Cancer including SDC2, TFPI2, WIF1, and NDRG4 methylation.

To elucidate the function of sodium pumps in CRC, Wu et al. performed immunohistochemical analyses of human CRC tissue specimens and established an orthotopic xenograft mouse model. They bring to light new mechanistical insights in the promotion of cancer cell metastasis. Elevated expression of the sodium pump α3 subunit promoted CRC liver metastasis *via* the PTEN/IGFBP3-mediated mTOR pathway. The glycoside bufalin could block the metastasis of CRC *via* this p53-dependent pathway.

In a cohort of 160 CRC patients, Qiao et al. demonstrated that the immunohistochemical expression of the chemokine CXCL7 and VEGF were correlated each other and significantly associated with N and TNM stage.

The use of diffusion-weighted imaging (DWI) for preoperative staging of rectal cancer is recommended by the European Society of Gastrointestinal Abdominal Radiology. Xia et al. demonstrated that a computed DWI post-process method, based on the calculation of random high b-value images, has a significantly higher image quality and diagnostic performance, compared with the conventionally acquired DWI.

Early detection of solid tumors of the liver and the pancreas and of gastroenteropancreatic neuroendocrine tumors (GEP-NET) is of paramount importance for the prognosis of the patients.

In patients with hepatocellular carcinoma (HCC), peripheral blood mononuclear cells show a specific DNA methylation signature, which can serve as a biomarker for the early detection of HCC and for its progression (8). Li et al. applied multiplex bisulfite sequencing to assess the methylation status of multiple CpG sites. In comparison with conventional methods, such as pyrosequencing, bisulfite-conversion-based methylation PCR, PCR cloning, or Sanger sequencing, this method allows effective large-scale analyses. In a cohort of 654 patients, the authors identified a minimal set of CpG sites that can predict the presence of HCC and. developed an individualized six-CpG-scorer-based nomogram as a potential non-invasive diagnostic tool for early HCC. However, external validation of this method is still lacking.

Serum level of carbohydrate antigen 19-9 is commonly used as a non in-invasive marker of pancreatic carcinoma; however, it often gives false positive results. In their paper, Cao et al. identified two metabolites the detection of which seems to predict with a high sensitivity and specificity the presence of stage-I pancreatic cancer. Although these findings were validated in an external cohort, the control group in the study did not include at-risk patients (e.g. those with chronic pancreatitis) and the sample size was relatively small.

In GEP-NET, the prognostic and diagnostic value of chromogranin A (CgA) level is controversial (9). Indeed, the elevation of CgA level is not specific to GEP-NET, as it is found in various other diseases. In their single-center retrospective analysis, Tsai et al. authors suggest that baseline CgA levels are associated with the disease extent and overall survival in GEP-NET patients.

The collection of articles herein presented ends with a review authored by Li and Chen, who discuss on the significant role of exosomes in the progression of HCC and on their potential clinical value as biomarkers and therapeutic targets.

In conclusion, the valuable papers published in this Research Topic have largely contributed to a critical discussion on the diagnostic advances in gastrointestinal tumors. It is our hope that the issues highlighted herein will drive further experimental inquiry ultimately leading to an improved clinical outcome in cancer patients in the future.

## Author contributions

All authors listed have made a substantial, direct and intellectual contribution to the work, and approved it for publication.

## Conflict of interest

The authors declare that the research was conducted in the absence of any commercial or financial relationships that could be construed as a potential conflict of interest.

## Publisher’s note

All claims expressed in this article are solely those of the authors and do not necessarily represent those of their affiliated organizations, or those of the publisher, the editors and the reviewers. Any product that may be evaluated in this article, or claim that may be made by its manufacturer, is not guaranteed or endorsed by the publisher.
